# Critical role of interleukin-33 in pathogenesis and progression of metabolic dysfunction-associated steatotic liver disease

**DOI:** 10.3389/fmed.2026.1762478

**Published:** 2026-03-31

**Authors:** Chunmei Ran, Xiaoyan Gao, Yongqin Zhou, Chibing Dai

**Affiliations:** 1 Department of Gastroenterology, Affiliated Renhe Hospital of China Three Gorges University, Yichang, China; 2 Hubei Key Laboratory of Tumor Microenvironment and Immunotherapy, College of Basic Medical Science, China Three Gorges University, Yichang, China

**Keywords:** diagnostic biomarker, hepatocellular carcinoma, inflammation, interleukin-33/suppression of tumorigenicity 2, metabolic dysfunction-associated steatotic liver disease, metabolism, pathogenesis, therapeutic target

## Abstract

Metabolic dysfunction-associated steatotic liver disease (MASLD), previously known as Non-alcoholic fatty liver disease (NAFLD) describes a diverse array of liver conditions linked to disrupted metabolic processes, ranging from hepatic steatosis and steatohepatitis to fibrosis, cirrhosis, and ultimately hepatocellular carcinoma (HCC). Currently, MASLD has progressively become a primary contributor to chronic liver disease globally. Due to its multifactorial etiology and varied clinical presentations, MASLD remains challenging in terms of accurate diagnosis and effective management. Accumulating evidence underscores the pivotal involvement of the interleukin-33 (IL-33)/suppression of tumorigenicity 2 (ST2) signaling pathway in critical aspects of MASLD pathophysiology, such as metabolic balance, oxidative stress response, intestinal barrier integrity, microbiota dynamics, inflammatory processes, hepatic fibrosis, and tumor development. IL-33 plays an exceptionally complex and central role in the pathophysiological processes of MASLD, and current evidence suggests that its actions may appear contradictory. Therefore, this review systematically integrates molecular insights into IL-33 biology and elucidates the regulatory networks underpinning IL-33/ST2 interactions, emphasizing their distinct roles at different MASLD stages. Furthermore, it is particularly noteworthy that IL-33 expression varies dynamically throughout disease progression. Additionally, the potential clinical utility of IL-33 as both a biomarker for early diagnosis and a therapeutic target is examined. By synthesizing fundamental and clinical research, this review aims to enhance the understanding of immunometabolic mechanisms involved in MASLD, thereby offering theoretical support and identifying future directions for personalized diagnostic and therapeutic approaches. The current research is largely limited to animal model studies, with a lack of longitudinal cohort studies in humans, which creates challenges for translational medicine.

## 1 Introduction

### 1.1 Metabolic dysfunction-associated steatotic liver disease

In modern society, the widespread intake of ultra-processed, sugar- and fat-dense foods, together with increasingly sedentary lifestyles, has placed individuals across all age groups at substantially heightened risk for metabolic dysfunction. As a result, the global rise in metabolic diseases and their associated complications has become a major focus of scientific investigation (1). NAFLD, recently redefined as metabolic dysfunction-associated steatotic liver disease (MASLD), encompasses a broad spectrum of liver conditions linked to metabolic impairment, ranging from simple steatosis to metabolic dysfunction-associated steatohepatitis (MASH), fibrosis, cirrhosis, and ultimately HCC (2). With the prevalence of MASLD, particularly its progressive form MASH, increasing worldwide, research on its pathogenesis, diagnosis, and treatment has intensified. Epidemiological data now identify MASLD as one of the most common causes of chronic liver disease globally, with incidence continuing to rise at an accelerating rate. Current estimates suggest a global MASLD prevalence of approximately 32% among adults, with higher rates in men (40%) than in women (26%). Considerable regional variation exists, likely reflecting differences in obesity prevalence, physical activity, fat distribution, socioeconomic conditions, and genetic predisposition. The highest prevalence is reported in South America (35.7%) and North America (35.3%), followed by Europe (30.9%) and Asia (30.0%), whereas Africa exhibits the lowest burden (13.5%). Notably, roughly 21% of individuals with MASLD progress to MASH (3). Although fewer than 10% of patients experience liver-related complications, the sheer scale of the affected population has made MASLD the fastest-growing contributor to liver-related mortality worldwide. In China, the prevalence has reached 29.2%, and both MASLD and MASH are projected to rise further in the coming years (4, 5). Globally, prevalence is predicted to increase to an estimated 55.4% by 2040 (6). As MASH and fibrosis advance, they markedly increase the need for liver transplantation, elevate the risk of liver cancer, and contribute to rising healthcare expenditures—highlighting the urgent need for integrated public health responses and multidisciplinary management strategies.

Diagnosis of MASLD requires confirmation of hepatic steatosis affecting more than 5% of hepatocytes, the presence of metabolic risk factors, particularly obesity and type 2 diabetes mellitus (T2DM), and the exclusion of significant alcohol intake (≥20 g/day for women and ≥30 g/day for men), alongside other chronic liver diseases (7). A major clinical challenge lies in identifying high-risk individuals within the expanding MASLD population. Current diagnostic tools—including imaging modalities and liver biopsy—have important limitations. Transient elastography and serum-based biomarkers, together with simple fibrosis assessment tools such as the MASLD fibrosis score, fibrosis-4 (FIB-4) index, and aspartate aminotransferase-to-platelet ratio index (APRI), are commonly used non-invasive options; however, their sensitivity for early-stage fibrosis remains insufficient. Although liver biopsy continues to be considered the “gold standard” for evaluating histological parameters such as steatosis, inflammation, ballooning, and fibrosis, its invasiveness and procedure-related risks limit its routine use for staging, monitoring, and therapeutic evaluation (4). Therefore, the development of more accurate, robust, and truly non-invasive biomarkers is urgently needed to improve early diagnosis, risk stratification, and fibrosis assessment. The heterogeneous etiology and diverse clinical manifestations of MASLD further complicate its treatment, with lifestyle modification remaining the foundational therapeutic strategy. The more recently proposed “multiple-hits” hypothesis suggests that MASLD arises from the interplay of numerous factors, including genetic predisposition, dietary habits, obesity, insulin resistance, dysregulated lipid metabolism, gut microbiota imbalance, oxidative stress (OxS), inflammatory mediators, and immune dysfunction (8, 9). Given the complex crosstalk between metabolic, inflammatory, and fibrotic pathways, single-target pharmacological approaches have achieved limited success in halting disease progression. As such, there is growing emphasis on developing therapeutic agents capable of modulating multiple pathogenic mechanisms while ensuring long-term safety and efficacy. Cytokines serve as key modulators of immune responses and possess diverse biological functions in both physiological and pathological conditions. Among these, interleukins constitute a major subgroup implicated in the development and resolution of liver diseases. Consequently, therapeutic strategies targeting interleukin signaling in hepatic disorders have emerged as a rapidly expanding area of research (10, 11).

### 1.2 Il-33

IL-33, belonging to the IL-1 cytokine superfamily, is encoded by a gene located at the 9q24 locus on human chromosome 9. IL-33 is initially synthesized as a precursor protein (proIL-33), which is predominantly located in the nucleus (12). Structurally, full-length IL-33 (fIL-33) is a 30 kDa protein lacking an N-terminal signal peptide and consisting of three separate domains: a nuclear localization domain at its N-terminus, a central domain sensitive to protease cleavage, and a cytokine activity domain at its C-terminal region (13). IL-33 is functionally versatile, acting as both a nuclear transcriptional modulator and a cytokine. Classified among “alarmin” molecules, IL-33 is actively secreted in response to damage at the cellular or tissue level (14).

**Figure 1:**
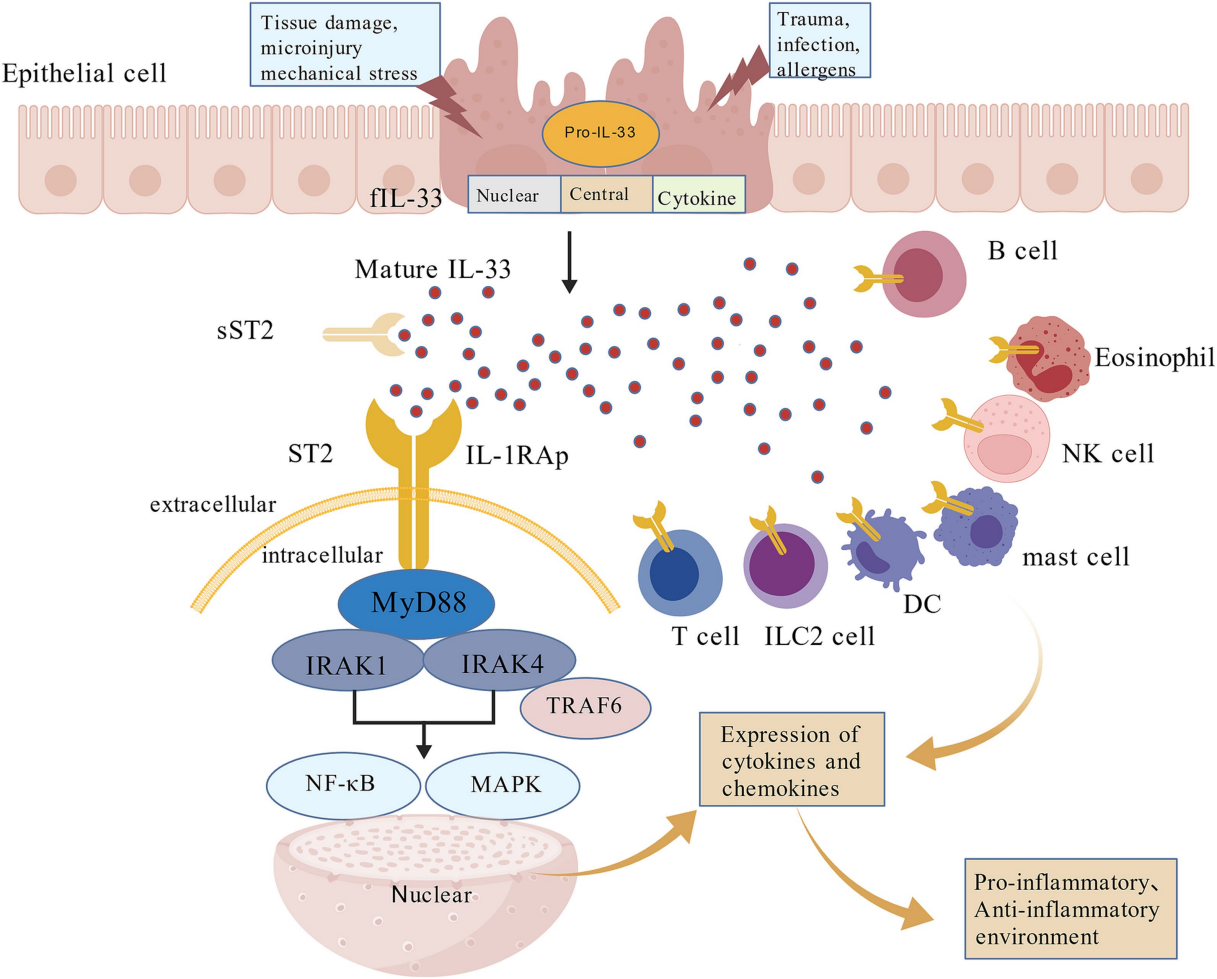
Structural characteristics, biological functions, and signaling pathways associated with IL-33. The proIL-33 is predominantly confined to the nuclear compartment. The fIL-33 is organized into three domains: an N-terminal nuclear localization domain, a central protease-sensitive domain, and a C-terminal cytokine domain. IL-33 exerts its immunomodulatory effects by signaling across various immune cell types. Through interaction with ST2, IL-33 triggers the NF-κB and MAPK pathways, resulting in transcriptional activation of genes involved in immune responses. In contrast, association with the sST2 inhibits this signaling process. MyD88, myeloid differentiation primary response protein 88; TRAF6, tumor necrosis factor receptor-associated factor 6; IRAK1/4, interleukin-1 receptor-associated kinase 1/4; NF-κB, nuclear factor kappa-light-chain-enhancer of activated B cells; MAPK, mitogen-activated protein kinase. Created with BioGDP.com (164).

The core feature of IL-33 signaling biology is that it is produced in an inactive precursor form, and upon cleavage by enzymes such as caspase-1, it attains its optimal activity. It then activates downstream signaling pathways through the ST2 receptor (12). IL-33 binds specifically to the suppression of tumorigenicity 2 (ST2) receptor complex, composed of ST2 and IL-1 receptor accessory protein, initiating signaling pathways via myeloid differentiation primary response protein 88(MyD88). This interaction activates the nuclear factor kappa-light-chain-enhancer of activated B cells (NF-κB) and mitogen-activated protein kinase (MAPK) signaling cascades. Conversely, soluble ST2 (sST2) serves as a decoy receptor that competitively inhibits IL-33/ST2 binding, thereby negatively modulating downstream signaling (12) (Figure 1). In the injury environment, in addition to the classical caspase-1 cleavage, passive release of necrotic cells or cleavage by other proteases (such as neutrophil elastase and cathepsin G) may produce active forms of IL-33. This “pathogenic enhancement” does not arise from the processing of the IL-33 protein into different subtypes but rather from abnormalities in its source, release, signaling, and the overall microenvironment. For example, although IL-33 is already present at high levels under homeostatic conditions, its expression can further increase during inflammation (such as in airway epithelial cells of COPD patients) (15). Inflammatory proteases from neutrophils and mast cells can process full-length IL-33 into shorter, mature forms (18–21 kDa) that contain the IL-1-like cytokine domain. These processed forms are 10 to 30 times more potent in activating mast cells and ILC2s compared to the full-length protein (16). Following its release into the extracellular milieu, the biological activity of IL-33 is constrained by multiple regulatory mechanisms. First, soluble forms of ST2 and IL-1RAcP function as decoy receptors that bind and neutralize IL-33 in biological fluids, thereby preventing its interaction with the membrane-bound receptor complex and attenuating downstream signaling (17). In addition, IL-33 undergoes rapid oxidative modification in the extracellular environment, during which cysteine residues form two disulfide bonds, leading to conformational changes in the IL-1-like cytokine domain and consequent loss of biological activity (18). The IL-33-ST2 signaling pathway is also inhibited by the single immunoglobulin domain IL-1R-related molecule (SIGIRR), which disrupts the ST2 and IL-1RAcP dimerization. The ubiquitin-proteasome system also inhibits this pathway by degrading ST2 (19). Additionally, nuclear-localized IL-33 displays context-dependent functions in HCC, exerting antitumor activity by repressing programmed death-ligand 1 (PD-L1) expression through interferon regulatory factor 1 (IRF1) degradation. However, SUMOylation-modified IL-33 promotes IRF1 stabilization, resulting in elevated PD-L1 and interleukin-8(IL-8) expression, immune escape, and enhanced tumor progression (20).

IL-33 exerts highly lineage-selective regulatory effects on diverse immune cell populations through its interaction with the receptor ST2. It functions as a central activator of group 2 innate lymphoid cells (ILC2s), directly promoting their proliferation and rapidly inducing the production of interleukin-5 (IL-5) and interleukin-13 (IL-13). These cytokines amplify type 2 immune responses and facilitate barrier tissue repair. Mast cells, eosinophils, and basophils are also highly responsive to IL-33; ST2-mediated signaling in these cells triggers degranulation and robust secretion of Th2-type cytokines, thereby establishing a type 2–dominant immune microenvironment within mucosal and barrier tissues and contributing to disorders such as asthma and allergic inflammation (21, 22). Within the adaptive immune system, IL-33 is a pivotal in regulating tissue-resident ST2^+^ regulatory T cells (Tregs) (23). Through both direct signaling and an indirect pathway involving dendritic-cell-derived interleukin-2(IL-2), IL-33 drives the expansion, functional stabilization, and enhanced suppressive capacity of these Treg subsets (24). This mechanism is essential for maintaining tissue homeostasis, resolving inflammation, and modulating metabolic processes. In certain infectious contexts and within tumor microenvironments, IL-33 can synergize with cytokines such as interleukin-12 (IL-12) or interferon-*γ* (IFN-γ) to potentiate the effector functions of natural killer (NK) cells, invariant natural killer T (iNKT) cells, and T helper 1 (Th1)/CD8^+^ T cells (25), underscoring its highly context-dependent immunomodulatory nature (13, 26, 27). Overall, IL-33 orchestrates a multilayered immune regulatory network characterized by strong tissue specificity and microenvironmental dependence. By directly activating tissue-resident immune subsets with high ST2 expression and integrating communication among dendritic cells, ILC2s, Tregs, and other immune cell populations, IL-33 functions as a critical molecular hub that links tissue stress signals to the reprogramming of immune responses.

IL-33 was first characterized as a cytokine capable of promoting type 2 immune responses through its activation of T helper 2 (Th2) cells and mast cells. Subsequent research has demonstrated that its functional scope is considerably broader. IL-33 potently activates ILC2s, Tregs, Th1 cells, CD8^+^ T lymphocytes, and NK cells, thereby influencing diverse physiological and pathological processes, including tissue regeneration, metabolic homeostasis, antimicrobial defense, inflammatory signaling, tumor progression, and neurological disorders (26). IL-33 is broadly distributed across multiple tissues, with its expression levels and biological effects varying according to tissue type and local physiological or pathological conditions. Under normal homeostatic states, IL-33 is primarily sequestered in the nucleus and is released as an “alarmin” in response to cellular damage or inflammatory stimuli. In epithelial tissues, such as the airway and intestinal mucosa, IL-33 activates immune cells and drives Th2-skewed immune responses, contributing to diseases such as asthma and inflammatory bowel disease (28). IL-33 is also abundantly expressed in endothelial cells, where it modulates vascular permeability and promotes inflammatory cell recruitment,being critically involved in the development of cardiovascular disorders (29). In fibroblasts, particularly those residing in the lungs and skin, IL-33 expression increases markedly during tissue repair and fibrotic remodeling. By enhancing fibroblast proliferation and amplifying inflammatory pathways, IL-33 contributes to the progression of idiopathic pulmonary fibrosis, intestinal fibrosis, and hepatic fibrosis (30). Elevated IL-33 levels in adipose tissue further indicate its potential as a biomarker for metabolic dysfunction (31). Moreover, IL-33 production by neurons and glial cells implicates it in neuroinflammation and neural repair. Dysregulation of the IL-33/ST2 axis has been observed in neuropsychiatric disorders, including depression, bipolar disorder, and schizophrenia, suggesting IL-33 may serve as a promising immune-related indicator of disease activity (32). Within tumor microenvironments, IL-33 can be secreted by malignant cells or tumor-associated fibroblasts, where it facilitates angiogenesis, immune escape, and tumor progression (33).

In the digestive system, aberrant IL-33 signaling contributes to various pathological processes. In the liver, IL-33 is produced by liver sinusoidal endothelial cells, hepatic stellate cells, cholangiocytes, intestinal epithelial cells, and several other cellular sources (34–37). In the liver, IL-33 plays multifaceted roles, with accumulating evidence indicating its involvement in MASLD, viral hepatitis, hepatic fibrosis, cirrhosis, and HCC (38, 39). Although its precise role in MASLD pathogenesis remains incompletely defined, current findings suggest that IL-33 influences disease progression through multiple mechanisms, including regulation of metabolism, modulation of inflammatory injury, maintenance of intestinal barrier integrity, promotion of fibrogenesis, and facilitation of tumor development. Elucidating the molecular mechanisms by which IL-33 signaling drives MASLD pathophysiology is expected to provide a theoretical foundation for the development of novel therapeutic strategies targeting this pathway.

Unlike classical pro-inflammatory mediators such as TNF-*α* and IL-1β, which predominantly orchestrate canonical inflammatory responses, IL-33 initiates a type 2 immune response with reparative potential during early tissue injury, yet transitions into a pivotal driver bridging fibrosis and HCC during the chronic persistent phase. Thus, IL-33 is not merely another member of the pro-inflammatory cytokine network. At the initiation stage, metabolic injury directly induces IL-33 release. Distinct from the direct cytopathic effects of viruses or toxins, the unique injury pattern in MASLD—characterized by hepatocellular lipotoxicity and oxidative stress—directly stimulates hepatocytes and liver sinusoidal endothelial cells to release IL-33, constituting a critical upstream event in disease onset. At the propagation stage, IL-33–activated type 2 immune responses, within the context of chronic metabolic dysregulation, trigger a cascade of inflammatory reactions. At the effector stage, IL-33 drives the characteristic pathological outputs of MASLD. On the one hand, it promotes metabolic fibrosis by activating hepatic stellate cells and enhancing collagen deposition; on the other hand, it shapes an immunosuppressive microenvironment enriched in regulatory T cells, thereby establishing a permissive niche for hepatocarcinogenesis.

**Figure 2:**
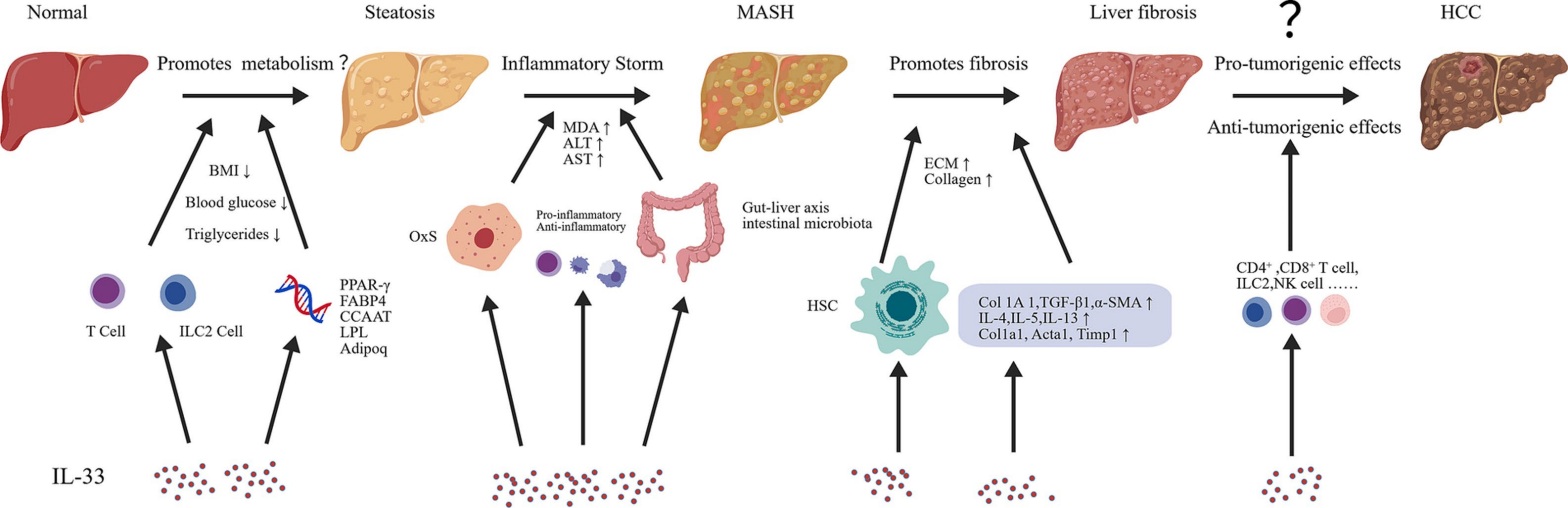
A dynamic model of functional switching of IL-33 during the progression of MASLD. As MASLD progresses to MASH, fibrosis, and HCC, the net effect of IL-33 shifts from protective to pathogenic. This transition is driven by changes in its proteolytic processing, cellular sources, and signaling context. Steatosis stage (promote metabolism): In the early phase of disease, hepatocytes or liver sinusoidal endothelial cells (LSECs) release a protective isoform of IL-33, primarily generated through caspase-1–mediated cleavage. This isoform signals through the ST2 receptor on immune cells such as ILC2s and Tregs, promoting anti-inflammatory responses and lipid clearance. Concurrently, it reduces the expression of lipogenesis-related genes, including PPAR-γ, FABP4, CCAAT/enhancer-binding protein (C/EBP), lipoprotein lipase (LPL), and adiponectin (Adipoq). MASH stage (inflammatory storm): With increasing hepatocellular oxidative stress and the influence of the gut–liver axis, IL-33 is converted into a pathogenic form. This form activates type 2 immune responses and triggers a destructive inflammatory cascade. Simultaneously, the protective ILC2/Treg axis becomes exhausted, shifting the immunological balance toward pathogenic dominance. Fibrosis and HCC stage: In advanced disease, activated hepatic stellate cells and tumor cells themselves may constitutively produce IL-33. On the one hand, IL-33 may exert context-dependent antitumor effects; on the other hand, it promotes fibrogenesis through mediators such as TGF-*β* and supports tumor growth via mechanisms including angiogenesis and maintenance of stem-like properties, thereby functioning as a key driver of end-stage liver disease. This model integrates stage-specific IL-33 dynamics, cell type–specific signaling pathways, and contextual microenvironmental cues to explain the dual and context-dependent roles of IL-33 in hepatic pathology. Created with BioGDP.com (164).

Collectively, this IL-33–centered axis in MASLD provides a fundamental theoretical rationale for therapeutically targeting the IL-33/ST2 pathway. Accordingly, this review proposes a central hypothesis: IL-33 is not a static mediator in MASLD progression but rather a stage-dependent regulatory factor whose biological effects dynamically shift across disease stages. In the early phase (transition from simple steatosis to MASH), IL-33 predominantly exerts protective and reparative functions; in the late phase (progression from MASH to fibrosis and HCC), it becomes a pathogenic driver. This functional switch is collectively determined by the intensity of metabolic stress, remodeling of the immune microenvironment, and dynamic regulation of ST2 receptor expression (Figure 2).

This study is a systematic narrative review, with all literature obtained through systematic searches. The databases searched include the PubMed/MEDLINE Core Collection. The search terms were constructed around core topics such as “IL-33” and “NAFLD/MASLD/NASH/MASH/HCC/liver fibrosis/gut-liver axis,” with the search time range spanning from database inception to December 2025. The inclusion criteria were original studies (both clinical and basic research) and high-quality reviews that clearly discuss the role of IL-33 in the development of MASLD and the prognosis of HCC. Conference abstracts and case reports were excluded. In the process of evidence synthesis and conclusion formation, we followed a clear prioritization: mechanistic studies preferred well-designed basic research, while clinical prognosis analyses focused on prospective cohort studies and large-sample retrospective studies. Studies with heterogeneous conclusions (e.g., due to differences in etiology or detection methods) were subjected to comprehensive analysis. This article aims for a qualitative synthesis and does not conduct quantitative meta-analysis, a limitation which is explicitly stated in the text.

## 2 Changes in IL-33 expression in MASLD

### 2.1 Clinical sample test results

Masoudreza et al. (40) conducted a study involving 97 patients with MASLD and found that circulating IL-33 concentrations increased significantly with advancing disease severity. Moreover, IL-33 levels were strongly correlated with key clinical indicators, including age, body mass index (BMI), blood lipid levels, liver function, and the extent of steatosis and fibrosis. Consistent with these findings, other studies have reported elevated IL-33 mRNA and protein expression in liver tissue, accompanied by increased serum IL-33 levels in patients with MASH (36). However, Hai et al. (41) presented contrasting results, observing increased intestinal IL-33 levels in MASLD patients but no significant alterations in hepatic IL-33 expression or its receptor ST2. Additionally, although circulating sST2 concentrations were markedly elevated in MASLD serum samples, IL-33 was undetectable in the same specimens. These discrepancies highlight the complex regulation and tissue-specific expression patterns of IL-33 in MASLD, suggesting that its role may differ depending on disease stage and the affected tissue. These contradictory findings may arise from three major sources: differences in sample selection, variability in detection methods, and genetic heterogeneity across populations. When selecting clinical samples for MASLD research, it is essential to account for disease stage, such as simple steatosis, MASH, and the presence or absence of metabolic syndrome comorbidities, as these factors can markedly influence IL-33 expression. Moreover, because most studies rely on samples from specific geographic regions, inter-population genetic variability may also contribute to inconsistent IL-33 expression patterns. In addition, methodological differences in tissue-based assays, including immunohistochemistry and ELISA, may result in substantial variability in measured IL-33 levels due to differences in sensitivity, specificity, and analytical procedures. Given that MASLD is a multifactorial disorder characterized by metabolic dysfunction, inflammation, and fibrosis, further studies are warranted to clarify how IL-33 expression varies across disease stages and how it contributes to disease progression.

### 2.2 IL-33 expression in mouse disease models

The role of IL-33 in the development and progression of MASLD has been demonstrated in multiple animal models. Experimental studies have demonstrated that after 24 weeks of high-fat diet (HFD) feeding, the mRNA expression of IL-33 and its receptor ST2 is significantly upregulated in both the serum and liver tissues of C57BL/6 J mice (36). Similarly, in the liver tissues of MASH C57BL/6 J mice fed an HFD for 20 weeks or a methionine–choline-deficient (MCD) diet for 10 weeks, both IL-33 and ST2 exhibited marked increases at the mRNA and protein levels (42). Another study using immunohistochemistry detected constitutive IL-33 expression in liver endothelial cells and showed that in C57BL/6 mice fed an HFD for 12 weeks, hepatic expression of IL-33, membrane-bound ST2, and soluble ST2 (sST2) was significantly elevated (43). However, conflicting evidence also exists. In one study, C57BL/6 mice fed an HFD for 24 weeks displayed significant upregulation of IL-33 and ST2 mRNA and protein in the intestine, whereas no notable changes were observed in the liver (41). In another obese mouse model, IL-33 mRNA and protein levels in visceral adipose tissue were higher in the HFD group compared with controls after 8 weeks, yet serum IL-33 levels remained unchanged between groups (44).

These findings indicate that IL-33 expression varies substantially across tissues and serum, possibly reflecting diet-induced metabolic changes, tissue-specific regulatory mechanisms, and environmental factors. Furthermore, inherent immunometabolic differences among mouse strains, such as C57BL/6 and BALB/c, contribute to distinct IL-33-mediated inflammatory and fibrotic responses. C57BL/6 mice, which exhibit a Th1-biased immune profile, are more prone to developing steatotic liver, hepatic inflammation, and fibrosis after 24 weeks of HFD feeding, accompanied by higher levels of IL-33, IL-13, and transforming growth factor-*β* (TGF-β) in liver homogenates. In contrast, BALB/c mice, characterized by a Th2-biased profile, develop more severe steatosis but display reduced hepatic inflammation and collagen deposition. These differences are closely associated with strain-specific immune cell compositions within metabolic tissues (45). Collectively, these findings support a potential role for IL-33 in MASLD pathogenesis; however, the precise causal relationships and underlying molecular mechanisms remain insufficiently defined and warrant further investigation.

## 3 Effects of IL-33 on the pathological process of MASLD

### 3.1 Metabolism

MASLD is a systemic metabolic disorder strongly associated with obesity, metabolic syndrome (MetS), inflammation in white adipose tissue, and triglyceride accumulation in the liver. These factors act synergistically to drive disease progression toward more advanced pathological stages, including steatohepatitis, fibrosis, cirrhosis, and HCC (46). MASLD is frequently regarded as the hepatic manifestation of MetS (47). Major risk factors include obesity, T2DM, dyslipidemia, hyperuricemia, and hypertension (48, 49). In particular, obesity leads to expanded adipose tissue mass and increased flux of free fatty acids (FFAs) to the liver, thereby exacerbating hepatic steatosis (50). Globally, the prevalence of MASLD, MASH, and advanced fibrosis is notably high among individuals with T2DM, reported at 55.5, 37.3, and 17.0%, respectively, underscoring T2DM as both a key risk factor and an accelerator of liver disease progression (51). As a metabolic modulator, IL-33 regulates adipogenesis, thermoregulation, and glucose homeostasis. Emerging evidence indicates that IL-33 levels increase in parallel with MetS severity and are strongly associated with major cardiovascular risk factors, including reduced high-density lipoprotein cholesterol (HDL-C) (Odd Ratio, OR = 5.34, 95% CI: 2.9–9.7, *p* < 0.01), elevated triglycerides (OR = 3.42, 95% CI: 1.9–6.1, *p* < 0.01), and hypertension(OR = 2.15, 95% CI: 1.1–4.1, *p* < 0.01) (52). Moreover, IL-33 concentrations correlate positively with dysglycemic markers such as hemoglobin A1c (HbA1c Spearman’s Rho, Rho = 0.558, *p* < 0.001) and insulin resistance (homeostasis model assessment of insulin resistance (HOMA-IR) Rho = 0.539, *p* < 0.001), as well as obesity-related parameters including body mass index (BMI Rho = 0.544, *p* = 0.013), visceral fat accumulation (VAT Rho = 0.371, *p* = 0.028), and hepatic lipid content(Rho = 0.350, *p* = 0.039) (53). Collectively, these findings suggest that IL-33 may serve as an early biomarker and a promising therapeutic target in both MetS and MASLD.

#### 3.1.1 Carbohydrate metabolism

IL-33 is recognized as a crucial regulator of glucose homeostasis through its influence on immune cell metabolism. In the pancreas, mesenchymal cell-derived IL-33 activates ILC2s, stimulating the secretion of stem cell factor 2 and IL-5. These cytokines upregulate aldehyde dehydrogenase expression in macrophages, leading to enhanced retinoic acid (RA) production. The increased RA subsequently acts on pancreatic *β* cells to promote insulin secretion and improve glucose clearance (54). In the liver, IL-33 drives ILC2-mediated secretion of IL-13, which directly suppresses gluconeogenesis in hepatocytes overexpressing hepatocyte nuclear factor 4α and glucose-6-phosphatase by activating the signal transducer and activator of transcription 3 (STAT3) pathway (55, 56). Moreover, IL-33 enhances glycolytic flux and oxidative phosphorylation in mast cells and facilitates glucose-dependent neutrophil recruitment. In contrast, ST2-deficient macrophages exhibit impaired glucose uptake, reduced lactate (LA) production, and enhanced mitochondrial activity following lipopolysaccharide (LPS) stimulation (57, 58). Importantly, LA amplifies glycolysis through hypoxia-inducible factor 1α (HIF-1α)-dependent signaling, whereas glycolysis itself inhibits ILC2 maturation by downregulating ST2 transcription, thereby establishing a negative feedback loop in IL-33 signaling (55, 59). IL-33-induced metabolic reprogramming also depends on the mammalian target of rapamycin complex, which enhances glycolytic activity in CD8^+^ T cells and ILC2s. Furthermore, by regulating peroxisome proliferator-activated receptor-*γ* (PPAR-γ) and diacylglycerol acyltransferase 1, IL-33 promotes both glucose and lipid metabolism (60). Additionally, some studies have demonstrated that IL-33 downregulates genes and proteins involved in adipocyte glucose uptake, lipogenesis, and lipid storage, such as glucose transporter 1/4(GLUT1/4), fatty acid-binding protein 4(FABP4), and peroxisome proliferator-activated receptor-*γ*(PPAR-γ), thereby reducing adipocyte glucose utilization and adipogenesis (53, 60).

In animal studies, obesity-induced overexpression of sST2 disrupts IL-33/ST2 signaling, resulting in a marked reduction of regulatory T cells (Tregs) and ILC2s in adipose tissue. This disruption contributes to the development of insulin resistance (61). Notably, exogenous administration of IL-33 has been shown to reverse obesity-associated impairments in ST2^+^ Tregs within visceral adipose tissue, attenuate adipose inflammation, and improve insulin sensitivity (62). Further experimental evidence indicates that elevated circulating sST2 levels are positively associated with insulin resistance and an increased risk of diabetes (63). Consistent with these findings, mice fed an HFD demonstrate significant improvements in fasting glucose levels, body weight, and insulin sensitivity following IL-33 treatment. In contrast, IL-33-deficient mice develop glucose intolerance and exacerbated insulin resistance (36, 55, 63).

#### 3.1.2 Lipid metabolism

In obesity, chronic inflammation, driven by proinflammatory cytokines (e.g., tumor necrosis factor (TNF-*α*)) and immune cells such as M1 macrophages and effector memory T cells, plays a central role in the development of insulin resistance and dysregulated lipid metabolism (64). Recent evidence highlights the pivotal role of IL-33 in maintaining adipose tissue homeostasis. IL-33 is predominantly produced by resident Lin^−^Sca-1^+^PDGFRα^+^podoplanin^+^ mesenchymal stromal cells. Obesity disrupts this homeostatic network by downregulating IL-33 expression and promoting the infiltration of proinflammatory immune cells, thereby amplifying metabolic inflammation (65). In obese rats, circulating IL-33 levels are markedly decreased, whereas sST2 concentrations are significantly increased. Although IL-33 expression is upregulated locally in the heart and adipose tissue, this compensatory response is largely counteracted by the inhibitory effects of sST2 (31, 61). Similarly, studies in HFD-fed mice have reported elevated IL-33 expression in visceral adipose tissue (44). However, De Oliveira et al. (66) suggested that despite IL-33 upregulation during obesity, its biological activity is impaired by multiple inhibitory mechanisms, ultimately failing to preserve adipose tissue homeostasis. In metabolically unhealthy obese individuals, IL-33 and ST2 expression are significantly increased and strongly correlated with key MetS indicators, including blood pressure, dyslipidemia, hepatic dysfunction, and systemic inflammation (67, 68). These findings suggest that the IL-33/ST2 axis may serve as a valuable biomarker of metabolic inflammatory status. The discrepancies among studies may be attributable to variations in sampling sites, as circulating IL-33 levels do not necessarily reflect its concentrations in adipose tissue. This divergence may stem from the constitutive expression of IL-33 by structural fibroblasts, endothelial cells, and epithelial cells across different human tissues.

Exogenous administration of IL-33 has been shown to alleviate obesity-associated metabolic disturbances. In HFD-fed mice, IL-33 treatment leads to significant reductions in body weight, fat mass, serum triglyceride levels, and adipocyte size. Notably, IL-33 expression is strongly correlated with leptin expression (62, 69). Mechanistically, IL-33 enhances insulin sensitivity by activating ST2^+^ ILC2s and Treg cells within adipose tissue. This activation promotes adipocyte proliferation and thermogenesis, thereby increasing energy expenditure and mitigating obesity progression (70, 71). In parallel, IL-33 downregulates lipogenic genes, including PPAR-*γ*, FABP4, CCAAT/enhancer-binding protein(C/EBP), lipoprotein lipase(LPL), and adiponectin(Adipoq) (44, 53). The lipogenesis-inhibiting effect of IL-33 also depends on activation of the canonical wingless/integrated *β*-catenin (Wnt/β-catenin) signaling pathway. Upon activation, this pathway suppresses the downstream transcription factor PPAR-γ, a central regulator of adipocyte differentiation. Consequently, IL-33 signaling inhibits the differentiation of preadipocytes and reduces lipid accumulation, thereby exerting a potent anti-adipogenic effect (44). In obese diabetic (ob/ob) mice, IL-33 promotes the accumulation of Th2 cells and drives macrophage polarization toward the anti-inflammatory M2 phenotype in adipose tissue, thereby attenuating obesity and lowering fasting blood glucose levels (72). Beyond its metabolic effects, IL-33 confers cardiovascular protection by inhibiting macrophage foam cell formation and cholesterol accumulation (66, 73, 74). Additionally, it promotes Th1-to-Th2 immune polarization, enhances fatty acid metabolism, and reduces both atherosclerosis and systemic lipid deposition (75–77).

In summary, these findings underscore IL-33 as a pleiotropic regulator of metabolic homeostasis. However, current evidence remains insufficient to support the notion that IL-33 exerts uniformly protective effects in metabolic disease. To date, most research has primarily focused on the effects of exogenous IL-33 administration on adipogenesis, while the role of endogenous IL-33 in obesity and related metabolic dysfunctions remains incompletely elucidated and lacks strong clinical validation. Further investigations are needed to determine whether metabolic diseases differentially regulate IL-33 expression at systemic versus tissue-specific levels, which may shed light on its context-dependent functions. Additionally, the dose-dependent effects of IL-33 may contribute to these contradictory findings, as its impact on metabolism and underlying mechanisms can vary with different administered doses. Moreover, the temporal relationship between IL-33 expression and the onset of metabolic dysfunction remains unclear. It is still uncertain whether IL-33 acts as a downstream mediator of metabolic disturbances or serves as an upstream driver of disease pathogenesis. Consequently, it is premature to conclude that IL-33 exerts a definitively protective effect on metabolic regulation. Prospective longitudinal studies are essential to establish a causal relationship between IL-33 dynamics and the initiation and progression of MASLD. Additionally, because most current studies have focused on obesity-related MASLD, the role of IL-33 in lean MASLD remains poorly understood and warrants further exploration.

### 3.2 OxS

OxS is a central mechanism in the initiation and progression of MASLD. Dysregulated lipid metabolism leads to the hepatic accumulation of FFAs, which induces lipotoxicity, impairs mitochondrial and endoplasmic reticulum function, and promotes excessive production of reactive oxygen species (ROS). Elevated ROS levels not only drive lipid peroxidation but also activate proinflammatory signaling pathways, such as NF-κB, thereby triggering inflammation, hepatocyte apoptosis, and disease progression from simple steatosis to MASH and ultimately liver fibrosis (78, 79). Emerging evidence suggests that IL-33 may amplify oxidative injury. Notably, IL-33 levels are negatively correlated with antioxidant enzyme activity, such as superoxide dismutase (SOD), and positively correlated with lipid peroxidation markers including malondialdehyde (MDA) and ROS. This indicates that IL-33 may exacerbate OxS by suppressing antioxidant defenses, thereby aggravating tissue injury (80). In advanced atherosclerosis, IL-33 has been shown to promote macrophage necrosis through increased OxS, as reflected by elevated intracellular 8-hydroxydeoxyguanosine and MDA levels, a reduced glutathione-to-oxidized glutathione ratio (GSH/GSSG), and diminished SOD activity (81). In the context of MASLD, IL-33 derived from intestinal epithelial cells (IECs) has been reported to induce gut microbiota dysbiosis and stimulate trimethylamine N-oxide (TMAO) production, which subsequently drives hepatic OxS and accelerates MASLD progression. Conversely, IL-33 deficiency attenuates microbiota-mediated TMAO synthesis and alleviates liver oxidative injury (41). TMAO, a gut microbiota-derived metabolite, has been implicated in promoting OxS, apoptosis, and mitochondrial dysfunction in cardiovascular disease (82). Collectively, current evidence suggests that IL-33 may aggravate MASLD progression by potentiating OxS. However, a new mechanism, namely oxidation-driven conformational changes, has been reported. The IL-33 molecule contains four free cysteine residues (Cys208, Cys227, Cys232, Cys259). When IL-33 is released as an alarmin from damaged cells into the extracellular environment, these cysteine residues are rapidly oxidized, forming two intramolecular disulfide bonds. This results in a dramatic conformational change in the IL-33 protein, leading to the loss of its biological activity (18). Therefore, this mechanistic link remains to be fully elucidated and warrants further validation through well-designed experimental and clinical studies.

### 3.3 Gut-liver axis

**Figure 3:**
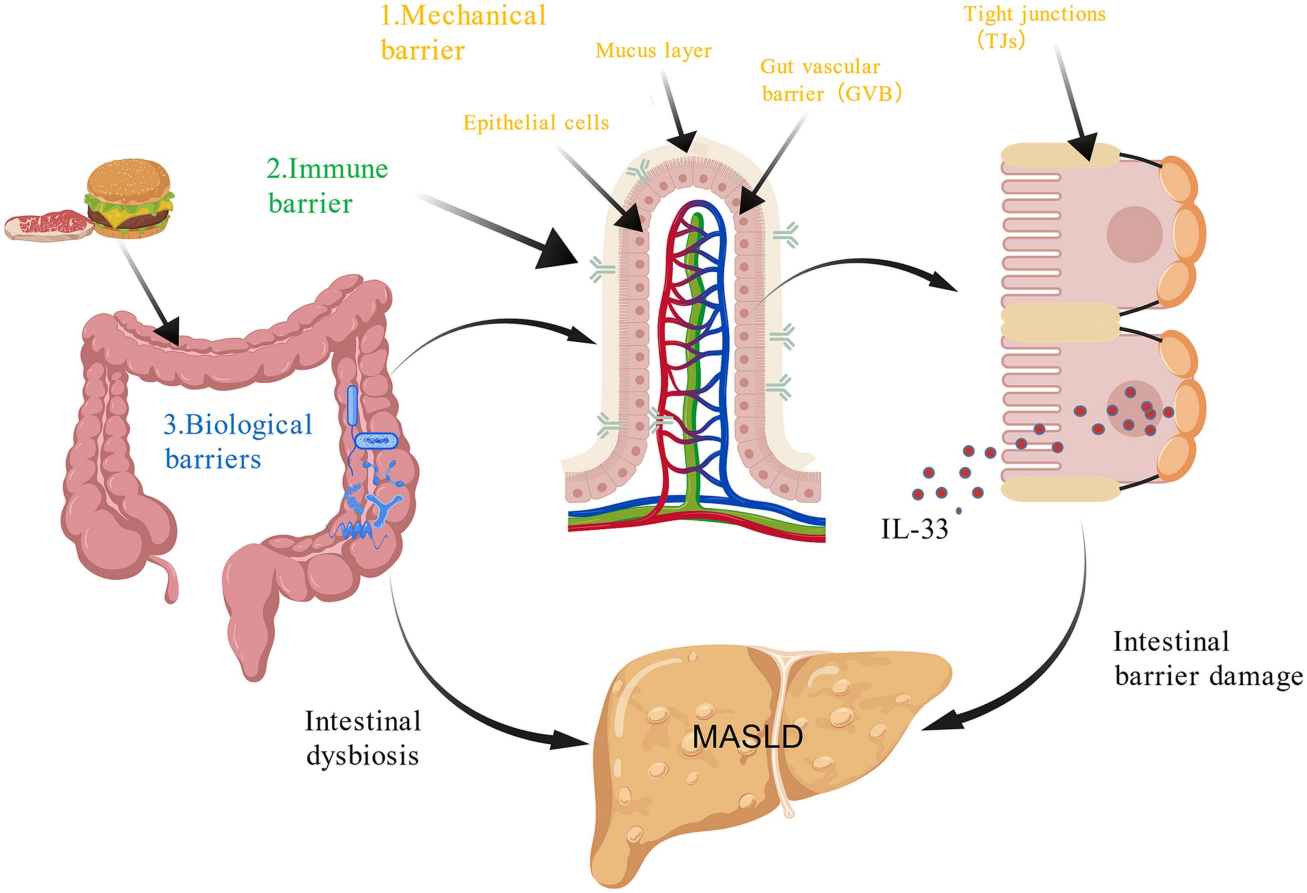
Intestinal dysbiosis and MASLD. 1. The gut mechanical barrier consists of multiple components: the mucus layer forms the outermost protective barrier; epithelial cells such as enterocytes line the intestinal wall; tight junctions (TJs), composed of specific proteins, regulate the paracellular transport, along with adherent junctions, desmosomes, and gap junctions; and the gut vascular barrier serves as the innermost defense layer. 2. The immune barrier comprises various immune cells predominantly situated in the intestinal mucosa, which also contains secretory immunoglobulin A. 3. The microbial barrier refers to the adult human microbiota, which contains approximately 100 trillion microorganisms, including commensal, pro- and anti-inflammatory, pathogenic, and nonpathogenic bacteria, as well as fungi and viruses, collectively contributing to the maintenance of intestinal homeostasis. Created with BioGDP.com (164).

The intestine and liver are functionally interconnected through the “gut-liver axis,” a bidirectional communication pathway that is central to the pathogenesis of MASLD. The development and progression of MASLD are not solely driven by hepatic metabolic dysregulation but are also profoundly shaped by the integrity of the intestinal barrier. This barrier is composed of three interdependent defensive layers, mechanical, immune, and biological, that work in concert to maintain intestinal homeostasis. When disrupted, intestinal permeability increases, microbial dysbiosis emerges, and the production of microbial metabolites becomes dysregulated. These alterations collectively contribute to hepatic injury and systemic metabolic disturbances (83) (Figure 3).

The role of IL-33 in maintaining intestinal homeostasis remains controversial. Several studies suggest that IL-33 may impair barrier integrity by downregulating TJ proteins, increasing intestinal epithelial permeability, and facilitating bacterial translocation, thereby amplifying inflammatory responses (84, 85). Epithelial injury itself induces the release of IL-33, which subsequently activates toll-like receptors (TLRs) on macrophages, leading to enhanced production of proinflammatory cytokines and further aggravation of epithelial damage. In parallel, IL-33 stimulates eosinophils to produce IL-13 and promotes Th9 cell proliferation, collectively impairing epithelial integrity, inhibiting tissue regeneration, and weakening mucosal immunity (86, 87). Recent evidence has also uncovered a dual regulatory effect of IL-33 on HIF-1α in both IECs and Th1 cells. Specifically, IL-33 interacts with nuclear HIF-1α in IECs and suppresses the transcription of downstream genes such as trefoil factor 3 (Tf3), cadherin 1 (Cdh1), and adenosine A2b receptor (Adora2b), thereby directly compromising epithelial barrier function. Concurrently, IL-33 enhances Th1 cell differentiation and interferon-*γ* (IFN-γ) production through the ST2-HIF-1α-T-box transcription factor 21 (Tbx21) axis, disrupting the Th1/Th17 balance and further promoting intestinal immune dysregulation (41).

In addition to its detrimental effects, IL-33 has also been shown to exert protective functions under specific conditions. It plays a direct and indirect role in repairing damaged epithelial layers, preserving intestinal barrier integrity, and mitigating inflammation associated with barrier disruption. Mechanistically, IL-33 induces the production of the antimicrobial peptide regenerating islet-derived protein 3 (REG3), which helps regulate commensal microbiota composition and suppress bacterial overgrowth. It also promotes the differentiation of IECs into goblet cells, facilitates wound healing, and attenuates inflammation by inducing M2 macrophage polarization (88, 89). IL-33 further activates group ILC2s to secrete amphiregulin (AREG), a member of the epidermal growth factor family. AREG binds to the epidermal growth factor receptor (EGFR) on IECs, thereby promoting tissue repair during inflammation and restoring mucosal integrity (90, 91). Consistent with this, exogenous IL-33 administration increases mucus production in mice, primarily composed of mucins, key structural components of the intestinal immune barrier (90). In parallel, recombinant IL-33 treatment has been found to downregulate the expression of ATP-binding cassette sub-family G member 5 and 8(ABCG5/8) genes in the colon. Given that ABCG5/8 are cholesterol transporters known to protect against TLR-mediated colonic inflammation, this finding suggests that IL-33 may influence lipid transport pathways to maintain intestinal homeostasis (92). Additionally, IL-33 enhances epithelial repair by upregulating miR-320 in IECs, thereby promoting epithelial cell proliferation (93). It also stimulates enterochromaffin cells (ECs) to release serotonin (5-HT), contributing to host defense and mucosal homeostasis (94). Beyond these effects, IL-33 recruits and activates innate immune cells to combat pathogens, amplifying type 2 immune responses that converge on tissue repair and barrier restoration following epithelial injury (95).

The intestinal microbiota serves as a crucial regulator of the gut-liver axis, with its metabolites, including short-chain fatty acids, bile acids, tryptophan derivatives, and TMAO, exerting profound effects on the development and progression of MASLD (96). An HFD disrupts intestinal barrier integrity, enabling the translocation of bacterial components such as lipoteichoic acid (LTA) into the liver, where they trigger the release of proinflammatory cytokines (e.g., IL-33), thereby amplifying hepatic inflammation and accelerating disease progression (97). Recent studies have further demonstrated that IL-33 derived from IECs promotes TMAO synthesis, leading to hepatic OxS injury and exacerbating the progression of MASLD. At the same time, IL-33 deficiency can reduce the synthesis of TMA (a precursor of TMAO) mediated by the gut microbiota and the subsequent induction of hepatic oxidative stress. IL-33 deficiency also leads to a significant reduction in gut bacteria capable of synthesizing TMA in MASLD mice (41). In a study by Arifuzzaman et al. (98), an inulin-rich diet was shown to reshape the intestinal microbiota and its metabolic profile, particularly bile acids, driving type 2 immune responses characterized by IL-33 release and ILC2 activation, ultimately contributing to MASLD pathogenesis. Another study also suggests that IL-33 exacerbates cholestatic liver injury and fibrosis caused by *Clonorchis sinensis* infection by regulating type II immune responses. This implies that IL-33 may contribute to liver damage by affecting bile acid metabolism (99). However, the relationship between IL-33 and the microbiota remains controversial. On one hand, IL-33 deficiency has been linked to microbial dysbiosis and the enrichment of proinflammatory bacterial taxa (100). On the other hand, other studies report that IL-33 deficiency improves microbial composition by increasing beneficial bacteria while reducing proinflammatory species (41). These discrepancies may stem from variations in experimental models, host genetic backgrounds, or baseline microbiome composition. In summary, a high-fat diet primarily disrupts intestinal homeostasis, leading to increased barrier permeability and dysbiosis, which facilitates the translocation of bacterial products (such as LPS) into the liver. These products activate liver immune cells (such as Kupffer cells) through the TLR4/NF-κB pathway, triggering sustained low-grade inflammation. This inflammatory state directly exacerbates hepatic insulin resistance and fat accumulation, while also activating hepatic stellate cells, driving fibrosis. At the same time, liver inflammation and metabolic alterations further exacerbate intestinal damage and dysbiosis through feedback mechanisms involving bile acids, thus forming a self-amplifying vicious cycle. Therefore, the gut is not only the initiating site of the disease but also a core driving force that continuously propels the pathological progression of liver disease.

Based on current evidence, IL-33 exhibits a dual role in the intestine. On one hand, it can aggravate MASLD progression by disrupting epithelial barrier integrity, triggering inflammatory responses, and altering microbial composition. On the other hand, IL-33 contributes to tissue repair and the maintenance of intestinal homeostasis. Overall, the mechanisms underlying IL-33 activity in the gut are highly complex and remain incompletely understood. These divergent findings are likely attributable to variations in experimental design, differences between acute and chronic inflammatory responses, disease stage, model selection, and host microbiome background. For example, subtle distinctions between IL33^−^/^−^ and ST2^−^/^−^ mice, as well as inter-individual variability in gut microbiota composition, may act as important confounding factors. Moreover, the biological effects of IL-33 appear to be stage-dependent. The timing and inflammatory context are critical in determining its role, making it essential to distinguish between acute and chronic inflammation. While IL-33 exacerbates disease in acute experimental colitis, it exerts a protective effect in chronic dextran sulfate sodium (DSS)-induced colitis (90, 101). Specifically, in mouse models, recombinant IL-33 administration worsens acute colitis through modulation of the amphiregulin/EGFR signaling pathway, whereas in chronic colitis it appears to confer protective effects. IL-33 promotes neutrophil infiltration during both acute and chronic phases; however, in the chronic stage, it can reduce the translocation of pathogenic bacteria across the damaged epithelium (90). Despite its protective roles in tissue repair, IL-33 has also been implicated in intestinal fibrosis and tumorigenesis during disease progression and associated complications (102, 103). These findings underscore the pivotal regulatory role of IL-33 in modulating immune responses across different disease stages in inflammatory bowel disease (IBD). To explain these stage-dependent effects, it is crucial to adopt a multifaceted investigative approach. In particular, further research into how IL-33 activity is shaped by post-transcriptional regulatory mechanisms may reveal its context-specific immunomodulatory functions under different inflammatory conditions.

The diverse effects of IL-33 likely stem from dynamic changes in the intestinal immune microenvironment, as well as underlying genetic and immunomodulatory factors that modulate its activity. This complexity underscores the need for further mechanistic studies to determine whether IL-33 primarily functions as a pathogenic driver or a protective regulator in MASLD. Importantly, future research should clearly distinguish the effects of exogenous IL-33 supplementation from those of endogenous IL-33 at different disease stages. Such insights will be critical for guiding the rational design of targeted therapeutic strategies.

### 3.4 Inflammation

The clinical spectrum of MASLD encompasses a continuum from simple hepatic steatohepatitis to MASH, the latter characterized by hepatocellular injury, inflammation, and progressive fibrosis (2). The onset of inflammation represents a critical watershed in the transition from steatohepatitis to Mash. IL-33 has been reported to exert both proinflammatory and anti-inflammatory effects in overweight and obese individuals. Several studies have demonstrated that circulating IL-33 levels are positively correlated with diastolic blood pressure, total cholesterol, serum aminotransferases (alanine aminotransferase(ALT) and aspartate aminotransferase(AST)), and markers of immune cell activation (67), suggesting a potential role in aggravating liver injury. Conversely, other findings indicate that IL-33 can significantly reduce ALT and AST levels and alleviate hepatocellular damage (63). In the context of obesity, chronic low-grade inflammation driven by immune cell infiltration into visceral adipose tissue plays a pivotal role in MASLD pathogenesis (104, 105). Experimental studies have shown that exogenous IL-33 administration reduces visceral fat mass, decreases adipocyte size, and attenuates adipose tissue inflammation (53). Mechanistically, IL-33 suppresses proinflammatory signaling [e.g., TNF-*α*, interleukin-1β(IL-1β), interleukin-6(IL-6)] by activating ILC2s and promoting type 2 immune responses, thereby inducing M2 macrophage polarization and mitigating obesity-associated inflammation (55, 72). These observations suggest that adipose tissue may utilize IL-33-mediated feedback mechanisms to maintain local immune homeostasis. Nevertheless, elevated IL-33 expression has also been consistently observed in patients with both acute and chronic liver diseases (106–108), raising the possibility that IL-33 may act as a pathogenic driver of hepatic inflammation under specific pathological conditions.

Like other members of the IL-1 cytokine family, IL-33 functions as both an inducer and modulator of inflammation and is abundantly expressed in adipose tissue and the liver (109). Under obese conditions, the immune landscape of adipose tissue undergoes profound remodeling, characterized by a decline in anti-inflammatory cell populations and an expansion of proinflammatory subsets, including CD44^+^CD62L^−^ effector memory T cells and M1 macrophages. These immune cells secrete inflammatory mediators such as TNF-*α* and IL-1β, which contribute to metabolic dysfunction (64). IL-33 acts as a potent chemoattractant for neutrophils at sites of inflammation (110, 111). Through engagement with its receptor ST2, IL-33 activates NF-κB and MAPK signaling cascades, driving the production of downstream cytokines, including IL-3, interleukin-4(IL-4), and IL-5, that amplify hepatic immune responses (109, 112). Furthermore, IL-33 promotes eosinophil infiltration via ILC2-derived IL-5, thereby exacerbating immune-mediated hepatitis and parenchymal injury (113). Hepatic sinusoidal endothelial cells (HSECs) are recognized as the primary source of IL-33, and cooperative interactions between activated macrophages and HSECs further potentiate its inflammatory effects (45). IL-33 release is also closely linked to NOD-like receptor family, pyrin domain containing 3(NLRP3) inflammasome activation, underscoring its role in sustaining chronic liver inflammation (39). For example, IL-33-primed neutrophil extracellular traps (NETs) can stimulate macrophages, promote NLRP3 inflammasome assembly, and induce the secretion of inflammatory factors such as IFN-*γ*, TNF-*α*, IL-1β, IL-6, monocyte chemoattractant protein-1(MCP-1), and granulocyte-macrophage colony-stimulating factor (GM-CSF) (81). Importantly, blockade of IL-33 signaling with neutralizing antibodies has been shown to suppress invariant NK T cell activation and ameliorate disease pathology, providing strong evidence for the pathogenic proinflammatory role of IL-33 in hepatitis (114).

In the liver, the functions of IL-33 are multifaceted, displaying either proinflammatory or anti-inflammatory properties depending on the pathological context and surrounding microenvironment (66, 115). These divergent effects may result from variations in experimental models, disease stage, or host-specific factors. Under physiological conditions, inflammatory responses triggered by disturbances in lipid metabolism are essential for maintaining systemic homeostasis and are tightly regulated to prevent excessive tissue injury. However, disruption of this regulatory equilibrium leads to the overproduction of lipid metabolites, proinflammatory cytokines, and adhesion molecules, thereby promoting the onset and progression of chronic metabolic disorders, including obesity, MASH, and atherosclerosis. IL-33 can be released in response to metabolic stress or cell death, amplifying inflammatory cascades and exacerbating both local hepatic injury and systemic metabolic dysfunction. Importantly, the pathogenic potential of IL-33 in inflammatory liver diseases appears to be closely linked to an imbalance between its regulatory and reparative functions. When IL-33-mediated immunoregulatory pathways, such as those involving Treg cells, ILC2s, and M2 macrophages, fail to adequately counteract its proinflammatory activity, IL-33 shifts toward a pathogenic role, thereby driving sustained inflammation and accelerating disease progression.

### 3.5 Liver fibrosis

Chronic liver injury arising from diverse etiologies can ultimately result in liver fibrosis and end-stage hepatic conditions, including cirrhosis and HCC. Liver fibrosis represents a wound-healing response to persistent hepatic damage and is characterized by the abnormal accumulation of extracellular matrix (ECM). Common initiating factors include viral hepatitis, excessive alcohol consumption, cholestatic disorders, autoimmune diseases, exposure to hepatotoxic agents, and MASH, all of which contribute to hepatocyte injury and structural remodeling of the liver (116). Importantly, liver fibrosis is recognized as a critical determinant of cardiovascular risk, cancer development, and overall survival in patients with MASH (117), emphasizing the importance of antifibrotic strategies as a central therapeutic objective. Hepatic stellate cells (HSCs) play a pivotal role in the initiation, progression, and potential reversal of liver fibrosis. In response to chronic injury, quiescent HSCs become activated and transdifferentiate into myofibroblasts, which secrete ECM components and perpetuate fibrogenic processes (117, 118). Notably, activated HSCs are a major source of IL-33, and the IL-33/ST2 signaling pathway has been implicated in both tissue regeneration and fibrotic remodeling following liver injury. During acute injury, IL-33 signaling promotes restoration of tissue integrity by enhancing fibrin deposition and wound closure. However, in the setting of prolonged or recurrent inflammation, this reparative mechanism becomes dysregulated, leading to excessive ECM deposition, a hallmark of liver fibrosis (119). Human and experimental studies have consistently demonstrated elevated IL-33 mRNA and protein levels in both liver tissue and serum samples of patients with MASH and cirrhosis, with expression levels positively correlating with fibrosis severity (34, 36, 120, 121). In murine models fed HFD or MCD diet, administration of IL-33 upregulated the expression of Col1A1 (a key mediator of fibrogenesis), TGF-*β*1 (a central pro-fibrotic cytokine), *α*-smooth muscle actin (α-SMA), and collagen (36).

Mechanistically, IL-33 promotes hepatic fibrogenesis primarily by inducing Th2 cell infiltration and stimulating the release of profibrotic cytokines such as IL-4, IL-5, and IL-13 (121). Among these, IL-13 is a key mediator. IL-13 directly drives HSC proliferation and collagen synthesis while simultaneously activating TGF-β signaling through the upregulation of matrix metalloproteinases, thereby accelerating ECM deposition (122–124). In addition, IL-13 upregulates the expression of key fibrotic genes, including *Col1a1*, *Acta1*, and *Timp1*, within HSCs. The profibrotic activity of IL-13 is critically dependent on galectin-3 (Gal-3), identifying Gal-3 as a potential therapeutic target, with Gal-3 inhibitors emerging as promising antifibrotic agents (42, 125, 126). IL-33 orchestrates these events by recruiting and activating Th2 cells, which in turn drive IL-13-mediated HSC activation and collagen deposition. This “IL-33-Th2-IL-13 axis” constitutes a central profibrotic pathway and provides a strong mechanistic rationale for the development of targeted immunotherapies in cirrhosis. Beyond Th2 cells, IL-33 also serves as a potent activator of hepatic ILC2s *in vivo*. Through the production of ST2-dependent IL-13, ILC2s further stimulate HSC activation, reinforcing their role as key drivers of liver fibrosis (127–129). Recent evidence additionally identifies IL-33 as a damage-associated molecular pattern. In murine models, IL-33 induces hepatocytes to secrete PC3-secreted microprotein (PSMP), a chemokine that promotes inflammatory macrophage activation and HSC stimulation via C-C motif chemokine receptor 2 (CCR2), thereby amplifying fibrogenic signaling and accelerating disease progression (130).

In summary, IL-33 is closely implicated in the pathogenesis of liver fibrosis, with elevated circulating and hepatic levels strongly correlating with disease severity and adverse clinical outcomes. These observations underscore its potential as a prognostic biomarker for fibrotic progression. Furthermore, the central role of the IL-33/ST2 signaling axis in driving hepatic fibrogenesis positions it as an attractive therapeutic target. A deeper understanding of the molecular mechanisms through which IL-33 regulates fibrotic remodeling will not only enhance insights into disease pathophysiology but may also pave the way for the development of innovative antifibrotic interventions.

### 3.6 Hepatocellular carcinoma

Without timely intervention, liver fibrosis and cirrhosis may progress to HCC, ultimately resulting in liver failure and the need for transplantation. HCC is the fourth leading cause of cancer-related mortality worldwide, and its incidence continues to rise. Chronic inflammation associated with viral hepatitis, alcohol-related liver disease, and MASLD is central to HCC pathogenesis (131). Notably, the incidence of MASLD-associated HCC has increased substantially, with more severe forms of MASH and advanced fibrosis markedly elevating the risk (132). In fact, MASLD has become the third most common underlying cause of HCC in the United States and is also linked to a higher likelihood of extrahepatic malignancies, including colorectal cancer (133, 134). The role of IL-33 in tumor biology is complex and remains a subject of debate. This dual-function cytokine has been implicated in both tumor promotion and suppression, not only across different cancer types but also within studies of the same tumor entity. In HCC, several reports have documented elevated IL-33 expression in both serum and tumor tissues, with higher levels correlating with advanced disease stage and poorer overall survival (135, 136) (Table 1). Conversely, other studies have observed significantly reduced IL-33 expression in HCC tissues, with lower levels also associated with unfavorable clinical outcomes (137, 138). A recent meta-analysis of 37 studies further demonstrated that increased IL-33 expression, whether in circulation or within tumors, was strongly associated with advanced tumor stage and grade, distant metastasis, greater tumor burden, and reduced 3- and 5-year survival rates (139). Collectively, these findings suggest that IL-33 may serve as a valuable prognostic and diagnostic biomarker in HCC. Comprehensive characterization of IL-33 expression patterns in liver cancer is therefore crucial to elucidate its precise role in tumor development and progression.

**Table 1:** Comparison of the impact of interleukin-33/ suppression of tumorigenicity 2 axis on hepatocellular carcinoma prognosis in different studies.

Author	Years	Country	Research subjects	Relationship between tumors and the IL-33/ST2 axis	Prognosis	IL-33 source
Wei et al. (38)	2024	China	Human HCC	IL-33 expression is elevated in tumor tissue.	High IL-33 expression is associated with a better prognosis.	tissue
Yang et al. (137)	2016	China	Human HCC, cirrhosis, hepatitis, and healthy controls	Reduced IL-33 expression in tumor tissue	Low IL-33 expression is associated with poor prognosis.	tissue
Heinrich et al. (138)	2022	USA	Human HCC	IL-33 inhibits tumor growth.	High IL-33 expression is associated with a better prognosis.	tissue
Jin et al. (144)	2018	China Singapore	Murine HCC	IL-33 inhibits tumor growth.	High IL-33 expression is associated with a better prognosis.	tissue
Wang et al. (20)	2023	China	Human HCC Murine HCC	IL-33 expression is reduced overall in tumors, with increased nuclear localization.	High IL-33 expression is associated with a better prognosis.	tissue
Stefan et al. (145)	2015	Germany	Human HCC	IL-33 expression is elevated in tumor tissue.	High IL-33 expression is associated with a better prognosis.	tissue
Yamagishi et al. (97)	2022	Japan	Murine HCC, Human HCC specimens	IL-33 levels are positively correlated with HCC.	Poor prognosis	tissue
Wang et al. (108)	2012	China	Human CHC	IL-33 levels are positively correlated with liver damage.	Poor prognosis	Serum
Wang et al. (135)	2020	China	Liver cancer cells, Murine HCC	Exogenous IL-33 promotes HCC growth.	Poor prognosis	Exoge-nous
Askoura et al. (136)	2022	Egypt	Human HCC	IL-33 levels are positively correlated with HCC.	Poor prognosis	Serum + tissue
Shen et al. (165)	2018	China	Human HCC, Healthy controls	Reduced IL-33 expression levels	Low IL-33 expression is associated with poor prognosis.	Serum
Bergis et al. (166)	2013	Germany	Human HCC	sST2 levels are positively correlated with HCC.	Poor prognosis	Serum
Pan et al. (159)	2020	China	Human HCC, Healthy controls	IL-33 genotype shows no significant association with HCC, whereas the ST2 genotype is positively correlated.	-	peripheral blood DNA
Wei et al. (160)	2018	China	Human HCC, Healthy controls	IL-33 genotype shows no significant association with HCC, whereas the ST2 genotype is positively correlated.	Poor prognosis	Serum
Zhao et al. (155)	2019	China	Human HCC	IL-33 expression is elevated in tumor tissue.	Poor prognosis	tissue
Zhang et al. (146)	2012	China	Human HCC, Healthy controls	IL-33 levels are positively correlated with HCC.	Poor prognosis	Serum + tissue
Tang et al. (154)	2024	China	Human CHB、HCC, Healthy controls	sST2 levels are positively correlated with HCC.	-	Serum
Xie et al. (156)	2019	China	Murine HCC, Human hepatocytes	IL-33 levels are positively correlated with HCC.	Poor prognosis	tissue
Huang et al. (167)	2025	USA	Human HBV Murine HBV	IL-33 levels are positively correlated with HCC.	Poor prognosis	Serum + tissue

The potential mechanisms underlying the antitumor effects of IL-33 have been increasingly explored. ILCs are key regulators of tumor immune surveillance, and their distribution varies among intra-tumoral, peri-tumoral, and non-tumoral liver tissues. IL-33 has been shown to promote the expansion of ILC2s, a subset associated with improved survival, suggesting that cytokine-mediated modulation of ILC composition may influence HCC prognosis (138). Additional evidence indicates that endogenous IL-33 can enhance antitumor immunity by either restricting Treg expansion or promoting the activity of tumor-infiltrating ILC2s (140, 141). In support of this, Amisaki et al. (142) demonstrated that IL-33-induced ILC2 activation facilitates the formation of tertiary lymphoid structures (TLS), which are ectopic lymphoid aggregates that develop in chronically inflamed tissues, including tumors. TLS formation has been linked to enhanced antitumor immune responses in a murine pancreatic cancer model. IL-33 also enhances eosinophil effector molecule expression, thereby promoting eosinophil-mediated tumor cell killing (143). Moreover, IL-33 has been reported to promote T cell activation and induce IFN-*γ* secretion, contributing to tumor suppression (144). However, IL-33 exhibits dual, context-dependent effects. In HCC, nuclear SUMOylation of IL-33 has been shown to promote immune evasion by stabilizing the transcription factor IRF1 and increasing PD-L1 expression (20). In contrast, non-SUMOylated IL-33 released from the liver can enhance antitumor CD4^+^ and CD8^+^ T cell responses, augment cytotoxicity, inhibit tumor progression, and prolong survival (144, 145). Conversely, under certain conditions, IL-33 may facilitate obesity-associated HCC by activating ST2^+^ Treg cells within the tumor microenvironment and driving CD8^+^ T cell exhaustion (97, 135).

IL-33 may contribute to tumorigenesis and cancer progression through multiple mechanisms. Upon binding to its receptor ST2, IL-33 activates NF-κB and other downstream signaling pathways (146), leading to M2 macrophage polarization and extensive eosinophil infiltration (147, 148). Concurrently, IL-33 stimulates Treg and ILC2 activation, sustaining a chronic inflammatory microenvironment that favors tumor initiation. Through the nuclear IL-33-SMAD signaling axis, IL-33 also promotes epithelial cell proliferation, further accelerating inflammation-associated malignancies (149). Additionally, IL-33 upregulates TNF-*α* mRNA expression, which may enhance tumor cell proliferation and angiogenesis (135). IL-33 further facilitates tumor progression, angiogenesis, and metastasis by expanding the Treg population, promoting M2 polarization of tumor-associated macrophages, and directly inducing proliferation and migration of tumor-prone cells (150, 151). Eissmann et al. (152) demonstrated that IL-33 enhances tumor vascularization and invasiveness, thereby accelerating HCC progression by supporting tumor cell survival, migration, and immune evasion. Moreover, IL-33 drives tumor development through the induction of multiple growth factors, including fibroblast growth factor (FGF) and amphiregulin (AREG), pro-angiogenic factors such as vascular endothelial growth factor C (VEGFC), and the establishment of a Th2-dominated immune microenvironment (153). However, the role of IL-33 in HCC is context-dependent. Tang et al. (154) proposed that IL-33 may exert protective effects by activating Th2 cytokines and promoting M2 polarization, whereas sST2 may exacerbate HCC by inhibiting IL-33/ST2L signaling. sST2 thus functions not only as a potential diagnostic biomarker but may also contribute to HCC pathogenesis.

Evidence also indicates that IL-33 contributes to HCC development through multiple mechanisms. IL-33 facilitates the acquisition of stem-like properties in HCC cells via activation of the c-jun n-terminal kinase (JNK) signaling cascade (155). Chronic exposure to tobacco smoke has been shown to markedly upregulate hepatic IL-33 expression, activating the p38 MAPK pathway and promoting epithelial-mesenchymal transition and cancer stem cell (CSC)-like traits in hepatocytes. These changes enhance self-renewal, migration, and invasiveness, collectively facilitating hepatocarcinogenesis (156). Elevated IL-33 levels in fibrotic livers have also been associated with impaired antitumor immunity, primarily through suppression of NK cell populations and activity, as demonstrated in murine models (157, 158). Additionally, IL-33 exerts pro-tumorigenic effects via the gut-liver axis. Specifically, LTA, a gut microbiota-derived component, accumulates in the liver and induces IL-33 release from senescent HSCs through gasdermin D (GSDMD)-dependent pyroptosis, thereby promoting obesity-related HCC (97). From a genetic perspective, Pan and Wei et al. (159, 160) investigated IL-33 and ST2 polymorphisms in Chinese HCC patients and healthy controls, revealing that carriers of the ST2 rs3821204 CC genotype had increased susceptibility to HCC, whereas IL-33 rs7025417 was not significantly associated with disease risk. These findings highlight the potential importance of IL-33-related genetic variants in hepatocarcinogenesis. Nevertheless, further studies with larger cohorts integrating clinical and mechanistic analyses are warranted to clarify the precise contribution of IL-33 polymorphisms to HCC development.

Several clinical studies have explored the correlation between IL-33 expression and prognosis in HCC, but the conclusions show significant heterogeneity. This contradiction is not due to data errors but rather reflects the complexity and context-dependent nature of IL-33’s biological functions. The following structural factors collectively shape its prognostic value in specific clinical cohorts (Table 2). The primary factor leading to divergent conclusions is the underlying liver disease etiology. In HCC associated with hepatitis B or C virus (HBV/HCV) infection, a strong virus-specific immune background is central. In this context, IL-33 may enhance antiviral and antitumor immunity by activating effector cells such as CD8^+^ T cells. In contrast, in MASLD-associated HCC, the environment is characterized by low-grade chronic inflammation, metabolic dysregulation, and an immunosuppressive microenvironment. Here, IL-33 tends to drive fibrosis and promote tumor progression. Next, heterogeneity in detection methods and biological localization significantly influences the findings. Sample source: serum IL-33 levels mainly reflect systemic damage or inflammatory status, whereas tissue IL-33 expression (especially localization within tumor parenchyma) provides more insight into its local role in the tumor microenvironment. These two measures have different prognostic implications and should not be equated. Antibody specificity: Commercial antibodies used in different studies may recognize different antigenic epitopes of IL-33, different cleavage forms (such as precursor or mature forms), or different oxidation states. Key differences in intracellular localization: Nuclear IL-33 mainly exerts its non-classical transcriptional regulatory function or serves as a storage form, while cytoplasmic or extracellular IL-33 is the key form that exerts its classic “alarmin” function and activates the ST2 signaling pathway. Most studies fail to differentiate the subcellular localization of IL-33, missing this potentially critical factor. Finally, the regulation of tumor biology and treatment background: The role of IL-33 may dynamically evolve during disease progression, and the timing of sample collection (e.g., at diagnosis, after neoadjuvant therapy, or at recurrence) is crucial. For instance, after treatments such as transarterial chemoembolization (TACE) or radiofrequency ablation, significant tumor necrosis may release large amounts of IL-33, and the levels detected at this point may reflect the treatment response rather than the inherent tumor biology, confounding its value as a baseline prognostic biomarker. Collectively, these factors indicate that the prognostic significance of IL-33 in HCC is highly context-dependent, shaped by disease etiology, detection strategy, subcellular localization, tumor stage, immune landscape, and treatment status, rather than representing a universal biomarker.

**Table 2:** Sources of heterogeneity in the prognostic value of IL-33 in hepatocellular carcinoma.

Impact dimension	Variable	Prognostic association of high IL-33	Main biological or technical explanation	Potential impact on study conclusions
Underlying liver disease etiology	HBV/HCV-related HCC	Often associated with improved OS	In virus-driven immunogenic contexts, IL-33 may enhance CD8^+^ T cell–mediated antiviral and antitumor immunity	Biases conclusions toward a protective role of IL-33
	MASLD/MASH-related HCC	Often associated with poor OS	In metabolic inflammation–dominated and immunosuppressive environments, IL-33 promotes type 2 immunity, fibrosis, angiogenesis, and Treg accumulation	Biases conclusions toward a tumor-promoting role of IL-33
Sample source	Serum IL-33	Highly inconsistent	Reflects systemic inflammation or liver injury rather than tumor-specific biology	Confounds tumor biology with disease severity
	Tumor tissue IL-33	More consistent but direction-dependent	More directly reflects local IL-33 sources, spatial distribution, and function within the tumor microenvironment	More suitable for mechanistic interpretation but sensitive to sampling location
Antibody and assay specificity	Total IL-33 detection	Marked variability in prognostic direction	Does not distinguish between precursor, mature, cleaved, or oxidized forms of IL-33	Different studies effectively measure different biological targets
	Isoform- or conformation-specific detection	Potentially clearer associations	Distinct IL-33 forms exert divergent nuclear regulatory versus extracellular signaling functions	Determines reproducibility and cross-study comparability
Subcellular localization	Nuclear IL-33	May associated with favorable prognosis	Functions in transcriptional regulation or represents an inactive storage form	Frequently overlooked in routine laboratory testing
	Cytoplasmic / extracellular IL-33	May associated with poor prognosis	Activates the ST2 signaling pathway, driving inflammation, immune suppression, and tumor progression	Represents the key biologically active alarmin form
Tumor stage and subtype	Early-stage HCC	May associate with improved prognosis	Antitumor immune surveillance remains intact and may be amplified by IL-33	Stage-dependent functional effects
	Advanced or aggressive HCC	Commonly associated with poor prognosis	Immune evasion dominates, and IL-33 supports protumor inflammation	Leads to opposite conclusions across cohorts
Treatment status and sampling timing	Treatment-naïve / at diagnosis	Pronounced heterogeneity	Reflects intrinsic tumor biology	Large baseline variability across cohorts
	Post-TACE / ablation / therapy	Prone to confounded associations	Passive IL-33 release from tumor necrosis reflects treatment response rather than tumor-intrinsic properties	Undermines its validity as a baseline prognostic biomarker

In summary, although the role of IL-33 in tumor biology remains controversial, accumulating evidence highlights its predominantly pro-tumorigenic effects in HCC. The dual functions of IL-33 appear to be highly context-dependent, influenced by both cytokine concentration and the composition of the tumor microenvironment. Notably, most studies to date have focused on the paracrine, cytokine-mediated actions of IL-33, whereas its cell-intrinsic, or autocrine, functions remain underexplored. To reconcile these divergent findings, it is critical to distinguish IL-33 derived from epithelial versus stromal compartments and to delineate its cell-autonomous versus cytokine-driven activities within malignant tissues. Future research should aim to dissect the functional heterogeneity of IL-33 and clarify the contributions of its distinct molecular forms. Such insights will be essential for the development of precision therapeutic strategies targeting the IL-33/ST2 axis in HCC.

## 4 Treatment prospects and challenges

### 4.1 IL-33/ST2 axis as a therapeutic target

The IL-33/ST2 signaling axis exhibits bidirectional regulation across different stages of MASLD, positioning it as a promising therapeutic target. In the early phases of metabolic dysregulation and MASLD, IL-33 may participate in protective feedback mechanisms that counteract immune imbalances induced by metabolic stress. Accordingly, strategies such as IL-33 mimetics, recombinant IL-33 protein, or ST2 receptor agonists could serve as novel immunometabolic interventions to mitigate metabolic and inflammatory disturbances. In contrast, during advanced stages of MASLD, including fibrosis and HCC, IL-33 contributes to hepatic fibrogenesis, sustains chronic inflammation, and promotes tumorigenesis, thereby accelerating disease progression. In these contexts, therapeutic approaches such as IL-33 neutralizing antibodies, soluble ST2-Fc fusion protein (sST2-Fc), or small-molecule ST2 antagonists may help inhibit fibrosis and tumor development, delaying further deterioration. Tailoring interventions according to disease stage will therefore be critical to achieving precision therapy.

### 4.2 Targeting downstream pathways

The IL-33 signaling pathway regulates liver metabolism and fibrosis through multiple downstream effectors, including IL-13, TGF-*β*, Gal-3, ILC2s, and gut-derived metabolites such as TMAO. Inhibitory or blocking strategies targeting these molecules have demonstrated therapeutic potential in animal models of MASLD. For instance, Gal-3 inhibitors reduce HSC activation and collagen deposition (42); IL-13R-directed cytotoxins significantly alleviate hepatic fibrosis and lower liver enzyme levels in MASH rats (161); and inhibition of TMAO production with 1,4-butanediol (DMB) attenuates OxS in HFD-fed mice (41). These findings suggest that combinatorial interventions targeting IL-33 alongside its downstream pathways may offer a novel and more effective therapeutic approach for MASLD, highlighting the potential of dual-target strategies for comprehensive disease control.

### 4.3 Biomarker potential

Alterations in IL-33 and its receptor expression in serum and tissues offer a promising avenue for the early diagnosis and therapeutic evaluation of MASLD. Circulating IL-33 and sST2 levels are closely linked to metabolic burden, hepatic inflammation, fibrosis stage, and hepatocarcinogenesis, rendering them valuable biomarkers for disease staging, prognostic assessment, and monitoring therapeutic responses (38, 67, 120, 137). Furthermore, the subcellular localization of IL-33 in hepatic tissue, whether nuclear or extracellular, as well as its expression patterns, may provide additional insights for pathological classification of liver diseases. Looking forward, integrating multi-omics approaches, including transcriptomics, proteomics, and metabolomics, to establish an IL-33/ST2 axis-based biomarker profile could enable dynamic and precise monitoring of MASLD progression and therapeutic efficacy.

A systematic comparison of existing MASLD biomarkers and therapeutic strategies helps clarify the clinical value, feasibility, and advantages of targeting IL-33. At the biomarker level, current indicators—including liver enzymes such as ALT and AST, fibrosis scoring systems such as FIB-4, and emerging serum biomarkers—are limited by insufficient stage specificity and an inability to dynamically reflect changes in the immune microenvironment. In contrast, IL-33 and its sST2 function as central mediators linking metabolic stress to immune responses. As such, they hold dual clinical value: early identification of MASH and prognostic assessment, including prediction of fibrosis progression and HCC risk. Moreover, serum detection techniques for IL-33/ST2 are well established (e.g., ELISA), with lower costs compared to imaging-based modalities such as FibroScan, making them suitable for implementation in primary healthcare settings. At the therapeutic level, current management strategies primarily rely on lifestyle intervention, which is often limited by poor adherence, and metabolic modulators such as Glucagon-Like Peptide-1(GLP-1) receptor agonists, which have relatively modest antifibrotic efficacy. Anti-inflammatory and antifibrotic agents, including the farnesoid X receptor (FXR) agonist obeticholic acid, are constrained by safety concerns such as pruritus and dyslipidemia. In contrast, IL-33–targeted therapy can be designed according to its stage-dependent biological effects, enabling a phased therapeutic strategy. In early MASLD, IL-33 agonists may activate protective type 2 immunity and promote tissue repair. In advanced disease, anti–IL-33 monoclonal antibodies or ST2 antagonists could block pathogenic signaling pathways—particularly IL-13–mediated hepatic stellate cell activation—thereby suppressing fibrogenesis. Combination with existing metabolic therapies may further enhance therapeutic efficacy. The principal advantage of this approach lies in its capacity to precisely modulate the immune–fibrosis axis, exerting both anti-inflammatory and antifibrotic effects. In addition, targeted delivery to hepatic stellate cells may reduce systemic exposure and associated risks.

From a feasibility perspective, anti–IL-33 monoclonal antibodies such as itepekimab have already advanced into late-stage clinical development for chronic obstructive pulmonary disease (COPD) (162), providing established experience in humanized antibody development and clinical application. Safety considerations remain important, as long-term IL-33 inhibition may theoretically increase susceptibility to infection; however, stage-specific short-term administration may help balance efficacy and safety.

In summary, IL-33 holds promise as both a biomarker and a therapeutic target in MASLD. As a biomarker, it may enhance the accuracy of noninvasive diagnosis and prognostic stratification. As a therapeutic target, stage-specific intervention strategies could enable precision modulation of disease progression. IL-33–centered approaches therefore represent a potentially transformative direction in MASLD management. Future priorities should include prospective cohort studies to validate its diagnostic and prognostic value, phase I clinical trials to evaluate antibody safety, and exploration of rational combination treatment strategies.

### 4.4 Outstanding questions and future directions

Although IL-33 has been proposed as a potential diagnostic biomarker for MASLD/MASH, its clinical utility has yet to be validated in multicenter studies with large patient cohorts. In the future, the development of simple and cost-effective IL-33 detection assays will be essential to enable widespread clinical application. Despite the promising therapeutic potential of targeting the IL-33/ST2 axis in MASLD, several challenges remain. First, precise modulation of IL-33 activity across different disease stages is critical to maximize therapeutic benefits while minimizing immune-related adverse effects, posing a significant hurdle for clinical translation. (1) *Disease stage-dependent treatment window and timing*: The timing of IL-33 pathway intervention is closely related to both efficacy and risk. In the stage of simple steatohepatitis, inflammation is mild, and the preventive intervention window is unclear, with the risk-to-benefit ratio facing significant challenges. The MASH and liver fibrosis stages are considered core treatment windows, as IL-33 is continuously released, driving the core processes of inflammation and fibrosis. Suppressing this pathway holds promise for dual inhibition. However, in the HCC stage, the situation is more complex—while inhibiting IL-33 may reduce background liver pro-tumor inflammation, it could also impair its potential anti-tumor immune surveillance function. Therefore, interventions must be based on precise tumor immune profiling to avoid unintended promotion of tumor progression. (2) *Intrinsic biological risks of pathway inhibition*: Inhibiting the IL-33/ST2 pathway is a “double-edged sword,” and its inherent physiological functions bring potential risks. IL-33 acts as an alarmin for tissue damage, and this pathway is central to type 2 immune responses and mucosal barrier defense. Additionally, IL-33 enhances the function of CD8^+^ T cells and NK cells in tumor immune surveillance. Systemic inhibition raises concerns about potentially weakening anti-tumor immunity and increasing the risk of tumor development. (3) *Additional risks posed by drug delivery strategies*: Using anti-IL-33 or anti-ST2 monoclonal antibodies carries the theoretical risk of forming immune complexes that could deposit in the liver, potentially exacerbating local inflammation. After long-term treatment, discontinuation may trigger a “rebound phenomenon,” inducing inflammation or fibrosis due to feedback regulation. More importantly, in the liver with existing potential precancerous lesions, altering IL-33 signaling could reshape the tumor immune microenvironment, inadvertently clearing tumor-suppressing immune cells, thereby creating favorable conditions for tumor growth. (4) *Advanced strategies for clinical translation*: To overcome the aforementioned challenges, next-generation intervention strategies are progressing toward precision and intelligent approaches. Subtype- or conformation-specific inhibitors (such as antibodies targeting only the active form of IL-33) aim to preserve some of its homeostatic functions, enhancing safety. Cell-targeted delivery systems, such as encapsulating siRNA or small molecule inhibitors in nanocarriers targeting activated HSCs, can achieve liver-specific drug enrichment, maximizing efficacy while minimizing systemic exposure. Rational combination therapies are another important direction, such as combining IL-33 pathway inhibitors with GLP-1 receptor agonists (to improve metabolism), or anti-fibrotic drugs, to achieve multi-pathway coordinated regulation. Currently, anti–IL-33 therapeutics have entered early-stage clinical development in several inflammatory diseases (Table 3). For example, the anti-IL-33 monoclonal antibody itepekimab has been evaluated in a phase II clinical trial involving patients with COPD (162). In parallel, anti–IL-33 monoclonal antibodies (e.g., etokimab) have entered clinical trials for atopic diseases such as atopic dermatitis (163), providing proof-of-concept evidence for the feasibility and preliminary efficacy of targeting this pathway. However, to date, no registered or completed pivotal clinical trials evaluating this class of agents in NAFLD or related liver diseases have been reported.

**Table 3:** List of IL-33-targeted drugs currently in clinical trials or already in practical application.

Drug name	Developer	Drug type	Mechanism of action	Main indications	Clinical stage	Key PubMed references
Itepekimab (REGN3500 / SAR440340)	Sanofi + Regeneron	Human monoclonal antibody	Neutralizes IL-33 and blocks IL-33/ST2 signaling	Asthma, COPD, Chronic rhinosinusitis	Phase II–III	(168, 169)
Tozorakimab (MEDI3506)	AstraZeneca / MedImmune	Human monoclonal antibody	Neutralizes IL-33 and inhibits ST2 and RAGE/EGFR signaling	COPD, Asthma, Atopic dermatitis	Phase I–III	(170, 171)
Etokimab (ANB020)	AnaptysBio	Humanized monoclonal antibody	Neutralizes IL-33	Atopic dermatitis (early trials)	Early clinical development	(163)

Based on robust preclinical evidence, it is reasonable to hypothesize that such agents may exert anti-inflammatory and antifibrotic effects in patients with NAFLD by suppressing ILC2 activity and reducing downstream cytokines such as IL-13. Nevertheless, their precise therapeutic efficacy in liver disease, optimal timing of intervention, long-term safety—particularly with regard to infection and tumor risk—and appropriate patient selection criteria remain to be definitively established through dedicated clinical trials. Second, the substantial metabolic and immunological heterogeneity among MASLD patients underscores the need for personalized intervention strategies tailored to individual variability. In addition, the differences between endogenous IL-33 and exogenously administered recombinant IL-33 in terms of signaling dynamics and biological effects remain incompletely understood, necessitating further investigation. Most current evidence derives from murine models, limiting direct extrapolation to humans. (1) *Microbial differences*: The composition and function of the human gut microbiota are far more complex than in experimental mice, and the microbiota is a key factor in regulating systemic inflammation and liver immunity. This may lead to a regulatory network of the IL-33 pathway in humans that differs from animal models. (2) *Immune differences*: There are inherent differences in the composition of immune cells in the human and mouse liver (such as innate lymphoid cell subpopulations and T cell pools), which may alter the final output of IL-33 signaling. (3) *Limitations of disease models*: The diet-induced mouse MASH model can simulate pathology within a few weeks, but it cannot replicate the chronic, multi-factorial course of human disease spanning decades, particularly failing to fully reflect the progressive evolution of the immune microenvironment during carcinogenesis.

To bridge these translational gaps, future research should focus on the following key unresolved issues and translational directions: (1) Longitudinal clinical cohort data: In prospective longitudinal MASLD cohort studies, systematically analyze the dynamic changes of serum active IL-33 and sST2, and rigorously correlate these with liver pathology (via serial biopsies) and clinical hard endpoints (decompensated cirrhosis, HCC occurrence), to define their evolutionary trajectory and predictive value across different disease stages (from simple fatty liver to MASH, fibrosis, and ultimately HCC). (2) There is a critical lack of experimental and clinical tools capable of discriminating distinct IL-33 isoforms and functional states. Current antibody-based assays typically measure total IL-33 without distinguishing nuclear versus extracellular localization, proteolytically processed forms, or redox-modified variants. Given that different IL-33 forms exert divergent, the development of isoform- and conformation-specific detection tools represents a key methodological priority for future studies. (3) The role of IL-33 across molecularly defined HCC subtypes remains largely unexplored. HCC is increasingly recognized as a heterogeneous disease comprising distinct molecular and immune subclasses. Whether IL-33 signaling differentially shapes tumor biology in immune-inflamed, immune-excluded, or metabolically driven HCC subtypes remains unknown. Integrating IL-33/ST2 profiling with transcriptomic and immunophenotypic stratification may uncover subtype-specific functions that are masked in bulk cohort analyses. (4) Future clinical trial designs targeting the IL-33/ST2 axis must explicitly account for tumor stage and disease context. In clinical drug trials, collect liver tissue before and after treatment to directly observe the impact of targeted interventions on key cells in this pathway (e.g., ILC2s, activated hepatic stellate cells) and downstream signaling. For precise interventions, future clinical trial designs must be stage-specific. For MASH/fibrosis stages, trials should focus on validating IL-33/ST2 inhibitors as anti-inflammatory and anti-fibrotic agents. In the HCC stage, the focus should shift to assessing their potential in combination with immune checkpoint inhibitors, with strict monitoring of potential risks.

In conclusion, therapeutic strategies targeting IL-33 in MASLD remain under investigation, and the long-term safety and efficacy of systemic IL-33 modulation in humans remain to be established. Addressing these gaps may facilitate the development of novel and more effective treatment strategies for MASLD.

## 5 Conclusion

In summary, IL-33 exerts complex and context-dependent effects on the onset and progression of MASLD, IL-33 influences a broad spectrum of processes, including glucose and lipid metabolism, hepatocellular injury, inflammatory responses, and the progression of liver fibrosis and HCC. In the early stages of MASLD, IL-33 primarily exerts protective effects by modulating metabolic pathways and inflammatory responses, highlighting its potential as an early biomarker and therapeutic target. However, as the disease advances to fibrosis or HCC, IL-33 may contribute to exacerbated inflammation, fibrosis, and tumorigenesis, reflecting its pathogenic role. This dual function of IL-33 is likely influenced by critical factors such as disease stage, local microenvironment, and dosage. Notably, IL-33 not only contributes to the early pathological events of MASLD but also plays a pivotal role in the transition to HCC. Future research should prioritize multicenter, large-cohort clinical studies to validate the diagnostic and therapeutic potential of IL-33. Simultaneously, it is crucial to comprehensively elucidate the dynamic regulatory mechanisms of the IL-33/ST2 axis across various disease stages, tissue-specific contexts, and systemic immune networks. Such mechanistic insights hold significant potential for advancing precision medicine approaches and guiding the development of targeted therapies for MASLD and its progression to HCC. Although this concept has been widely discussed, it remains severely limited by the lack of robust human data. Most existing evidence is derived from animal models or *in vitro* experiments, and has not yet been validated in large-scale, well-stratified human cohort studies. In particular, longitudinal investigations tracking serum and/or tissue IL-33 levels across distinct disease stages—and correlating these levels with histological progression and clinical outcomes—are still lacking. Consequently, the authenticity and generalizability of the proposed “stage-dependent effects” of IL-33 remain unconfirmed in human MASLD/MASH populations. This gap underscores a central knowledge deficit and a key limitation in our current understanding of the biological role of IL-33 in MASLD/MASH. Therefore, this hypothesis should be regarded not as an established conclusion, but rather as a critical research priority that warrants rigorous validation in future translational and clinical studies.
